# In vitro and in vivo protective efficacies of antibodies that neutralize the RNA N-glycosidase activity of Shiga toxin 2

**DOI:** 10.1186/1471-2172-11-16

**Published:** 2010-03-24

**Authors:** Kwang-il Jeong, Susan Chapman-Bonofiglio, Pradeep Singh, Jongo Lee, Saul Tzipori, Abhineet S Sheoran

**Affiliations:** 1 Department of Biomedical Sciences, Cummings School of Veterinary Medicine, Tufts University, Medford, MA 02155 USA; 2 Department of Mathematics, Southeast Missouri State University, Cape Girardeau, MO 63701, USA

## Abstract

**Backgound:**

Shiga toxin 2 (Stx2), one of two Stx liberated by Stx-producing *Escherichia coli*, is composed of an A subunit monomer and a B subunit pentamer, and is directly linked with hemolytic uremic syndrome in children. The pentameric B subunit binds to its cell surface receptor Gb_3 _for toxin internalization, and the A subunit follows intracellular retrograde transport to the cytosol where its RNA *N*-glycosidase activity (RNA-NGA) shuts down the protein synthesis, and leads to cell death. The present study investigated the ability of 19 Stx2 A subunit-specific human monoclonal antibodies (HuMAbs) to neutralize the RNA-NGA, and the association this neutralizing activity with protection of HeLa cells and mice against Stx2-induced death.

**Results:**

The HuMAbs that were stronger inhibitors of RNA-NGA were also better at neutralizing Stx2 mediated HeLa cell death, and those that were weaker inhibitors of RNA-NGA activity were also weaker in protecting HeLa cells. These results suggest that the ability of an A subunit-specific antibody to block the RNA-NGA of the toxin is directly related to its ability to neutralize Stx2-mediated HeLa cell death. However, with the exception of the best RNA-NGA blocking antibodies 5C12 and 2F10, the efficacies of antibody neutralization of RNA-NGA of Stx2 did not correlate with their *in vivo *protective efficacies. The HuMAb 6C3, which neutralized RNA N-glycosidase activity of Stx2 less effectively than the HuMAbs 6D8 and 6B7, protected 100% of the mice against Stx2 challenge at 50 μg/mouse dose. In contrast, the HuMAbs 6D8 and 6B7, which neutralized RNA N-glycosidase activity of Stx2 more effectively than 6C3, protected 20% and 0% mice at that dose, respectively.

**Conclusions:**

The neutralization efficiency of the RNA-NGA of Stx2 by A subunit-specific antibodies correlate strongly with their abilities to protect HeLa cells against Stx2-mediated toxicity but only the strongest RNA-NGA-neutralizing antibodies correlate very well with both protecting HeLa cells and mice against Stx2 challenge.

## Background

Infection with Shiga toxin (Stx)-producing *Escherichia coli *(STEC) is the most significant cause of hemolytic uremic syndrome (HUS), the leading cause of acute renal failure in children [1-4]. Two antigenically distinct Stx, Stx1 and Stx2, are associated with the development of HUS. Stx1 and Stx2 are similar in basic structure [5], binding specificity [5] and mode of action, but quite distinct in disease outcome [6]. Stx2-producing strains are more frequently associated with HUS in humans than Stx1- or both Stx1- and Stx2-producing strains [7,8].

The Stx molecule consists of an A-subunit monomer and a B-subunit pentamer [5,9,10]. The pentameric B subunit binds to its cell surface receptor CD77, also called globotriaosyl ceramide (Gb_3_; Galα1-4Galβ1-4glucosyl ceramide) [11,12] with the exception of Stx2e, which binds preferentially to globotetraosylceramide (Gb_4_; GalNAc β1-3Galα1-4Galβ1-4glucosyl ceramide) [13,14]. Internalized Stx is then delivered to the trans-Golgi network (TGN), where it is carried by retrograde transport to the endoplasmic reticulum (ER), and then to the cytosol [15,16]. During this process, the A subunit is nicked by the membrane bound furin protease, generating a catalytically active N-terminal A1 fragment and a C-terminal A2 fragment; both fragments remain linked by a disulphide bond [15,17]. The disulphide bond is subsequently reduced, and the active A1 component is released. The released A1 fragment has N-glycosidase catalytic activity and removes a specific adenine base from the 28S rRNA of the 60S ribosomal subunit [18,19]. Because this adenine base is on a loop of rRNA that is important for elongation factor binding, the toxin is able to shut down the protein synthesis and cause cell death.

We have recently produced human monoclonal antibodies (HuMAbs) against Stx1 and Stx2, and evaluated them in animal models for their efficacy against systemic challenge with the toxins [20,21]. We selected for further analysis 5C12, a Stx2 A subunit-specific HuMAb, based on its superior efficacy over others in protecting mice against lethal challenge with Stx2 and Stx2 variants [22]. Preclinical evaluation in a piglet model of infection has shown that 5C12 protects piglets against Stx2-induced fatal neurological symptoms, even when the antibody is administered well after onset of diarrhea and oral STEC challenge (48 hours post-challenge) [23]. In this model, diarrheal symptoms precede systemic complications associated with Stx2 uptake from the gut, as is observed in children.

The aim of the present study was to investigate whether 5C12 and other A subunit specific HuMAbs neutralize the RNA *N*-glycosidase activity (RNA-NGA) of the toxin, and to assess whether this inhibitory activity is indicative of an antibody's ability to neutralize Stx2 toxicity in vitro or in vivo.

## Results

### Grouping of the HuMAbs based on their strength to neutralize Stx2-mediated HeLa cell cytotoxicity

Overall, HuMAbs showed a dose-dependent neutralization of Stx2 (20 ng/ml), with maximum neutralization occurring at the highest antibody concentration of 10 μg/ml (Table 1). Based on the Stx2-neutralizing activity, the 19 HuMAbs analyzed in this study were assigned to high, medium or low neutralizing groups. The neutralizing activity mostly differed significantly between the antibodies of the three groups (Table 1). The HuMAbs 2F10, 3E9 and 5C12 neutralized Stx2-mediated HeLa cell cytotoxicity significantly better than all other antibodies, and therefore, were grouped as high neutralizing antibodies. They neutralized 89%-97% of the Stx2 induced HeLa cell cytotoxicity at the highest antibody concentration of 10 μg/ml. HuMAb 5C12 was especially potent since it neutralized about 90% toxicity of Stx2 even at 1.25 μg/ml. The Stx2-neutralizing activity of the medium neutralizing group (6H5, 6H7, 7C4, 9H9, 14C12, 5E12, 6D8, 6B7, 6C3, and 1G1) was lower than the high neutralizing group since it neutralized 45%-70% of Stx2-mediated HeLa cell cytotoxicity at the highest antibody concentration. The low neutralizing group included the remaining six HuMAbs (4H10, 6E6, 1G12, 3A2, 5F2, and 7F2) which showed minimal to mild toxin neutralization (<45%) even at the highest antibody concentration.

**Table 1:** Neutralization of Stx2-mediated HeLa cell cytotoxicity by Stx2 A subunit-specific HuMAbs.

Neutralization groups^1^	HuMAb	Percent neutralization (mean ± SD) at different HuMAb doses		
		
		10 μg/ml	1.25 μg/ml	0.156 μg/ml
High	5C12	97 ± 1.7^a^	89 ± 4.1^a^	73 ± 5.1^a^
	3E9	94 ± 3.3^ab^	80 ± 6.5^ab^	52 ± 5.3^ab^
	2F10	89 ± 4.6^b^	71 ± 7.6^b^	50 ± 4.2^b^
				
Medium	7C4	69 ± 6.5^c^	52 ± 6.2^c^	38 ± 4.1^c^
	6D8	68 ± 9.6^c^	48 ± 8.5^c^	38 ± 3.2^c^
	9H9	65 ± 10.6^c^	48 ± 7.4^c^	29 ± 5.9^cd^
	6H7	64 ± 7.1^c^	46 ± 6.5^c^	22 ± 4.5^d^
	14C12	63 ± 6.8^c^	39 ± 7.4^cd^	29 ± 8.8^cd^
	5E12	59 ± 9.0^cd^	36 ± 6.4^cd^	17 ± 4.6^de^
	6B7	57 ± 6.2^cd^	37 ± 5.5^cd^	23 ± 3.6^d^
	6H5	57 ± 5.9^cd^	39 ± 3.5^cd^	25 ± 3.9^d^
	6C3	50 ± 7.1^de^	37 ± 5.1^cd^	16 ± 3.6^de^
	1G1	46 ± 7.3^de^	27 ± 7.5^d^	15 ± 1.9^de^
				
Low	4H10	40 ± 8.5^ef^	13 ± 5.3^e^	10 ± 5.3^e^
	5F2	35 ± 4.7^f^	13 ± 5.5^e^	1 ± 0.7^f^
	6E6	34 ± 5.6^f^	25 ± 6.1^d^	9 ± 4.6^e^
	3A2	31 ± 8.1^fg^	15 ± 8.1^e^	14 ± 9.1^de^
	7F2	25 ± 4.7^g^	14 ± 7.8^e^	7 ± 5.1^e^
	1G12	15 ± 4.7^h^	11 ± 5.9^e^	10 ± 5.5^e^
				
Placebo control	IgG1κ	5 ± 3.8^h^	5 ± 1.7^e^	2 ± 1.5^f^

^1^The HuMAbs were assigned to high (5C12, 3E9 and 2F10), medium (7C4, 6D8, 9H9, 6H7, 14C12, 5E12, 6B7, 6H5, 6C3 and 1G1) or low (4H10, 5F2, 6E6, 3A2, 7F2 and 1G12) neutralizing groups based on their abilities to neutralize Stx2-mediated HeLa cell cytotoxicity.

^a-h^Values within a column with different superscripts are significantly (*P *< 0.05) different.

### Neutralization of RNA-NGA of Stx2 by the HuMAbs

**Figure 1:**
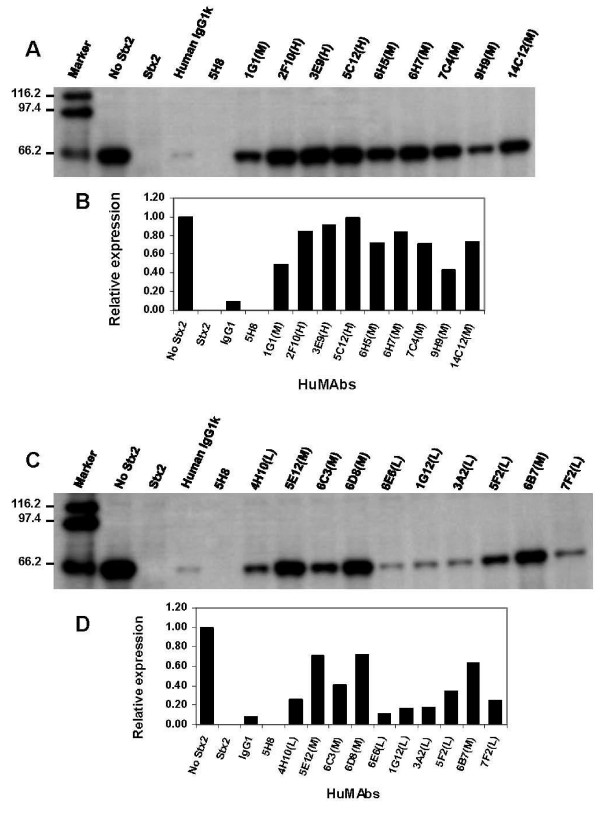
**Neutralization of the RNA-NGA of Stx2 by the A subunit-specific antibodies**. The letters 'H', 'M' or 'L' within parenthesis after a HuMAb designation represent high, medium or low HeLa cell cytotoxicity-neutralization groups of the antibodies, respectively. The HuMAbs 2F10, 3E9, and 5C12 were the strongest in blocking the RNA-NGA of Stx2 since visual inspection (Fig. 1A and 1C) and semi-quantitative densitometry analysis (Fig. 1B and 1D) of the luciferase bands showed that the amount of produced in the presence of these antibodies was similar to that translated in the absence of Stx2 ("no Stx2" lane). The luciferase band analysis also revealed that the neutralization of RNA-NGA of Stx2 by medium Stx2-cytotoxicity neutralizing group antibodies 6H5, 6H7, 7C4, 14C12, 5E12, 6D8 and 6B7 was moderate since relative expression of the luciferase bands was between 0.60 - 0.80. The expression of the luciferase bands in presence of the other 3 antibodies (9H9, 6C3 and 1G1) of the medium Stx2-cytotoxicity neutralizing group was weaker (relative expression of the luciferase bands between 0.40 - 0.60) than the other antibodies of this group but still better than that of the low Stx2-cytotoxicity neutralizing group antibodies. The expression of the luciferase bands in presence of the low Stx2-cytotoxicity neutralizing group antibodies (4H10, 6E6, 1G12, 3A2, 5F2, and 7F2) was very weak (relative expression of the luciferase bands <0.40). The experiment was repeated once with similar results.

The strongest Stx2-cytotoxicity neutralizing HuMAbs 2F10, 3E9, and 5C12 were also the strongest in blocking the RNA-NGA of Stx2 (Fig. 1). Visual inspection (Fig. 1A and 1C) and semi-quantitative densitometry analysis (Fig. 1B and 1D) of the Western blot bands showed that the amount of luciferase produced in the presence of these antibodies was closer to that translated in the absence of Stx2. The quantitative expression of the luciferase band in presence of the HuMAbs 2F10, 3E9, and 5C12 relative to when Stx2 was absent was 0.85, 0.92 and 0.99, respectively (Fig. 1B). The luciferase band analysis also revealed that the neutralization of RNA-NGA of Stx2 by 7 (6H5, 6H7, 7C4, 14C12, 5E12, 6D8 and 6B7) of the 10 medium Stx2-cytotoxicity neutralizing group antibodies was moderate (relative expression of the luciferase bands between 0.60 - 0.80). The expression of the luciferase bands in presence of other 3 antibodies (9H9, 6C3 and 1G1) of the medium Stx2-cytotoxicity neutralizing group was weaker (relative expression of the luciferase bands between 0.40 - 0.60) than the other antibodies of this group but still better than that of the low Stx2-cytotoxicity neutralizing group antibodies. The expression of the luciferase bands in presence of the low Stx2-cytotoxicity neutralizing group antibodies (4H10, 6E6, 1G12, 3A2, 5F2, and 7F2) was very weak (relative expression of the luciferase bands <0.40). Overall, the strength by which the HuMAbs neutralized RNA-NGA of Stx2 correlated with their strength to neutralize Stx2-mediated HeLa cell cytotoxicity (Fig. 1).

**Figure 2:**
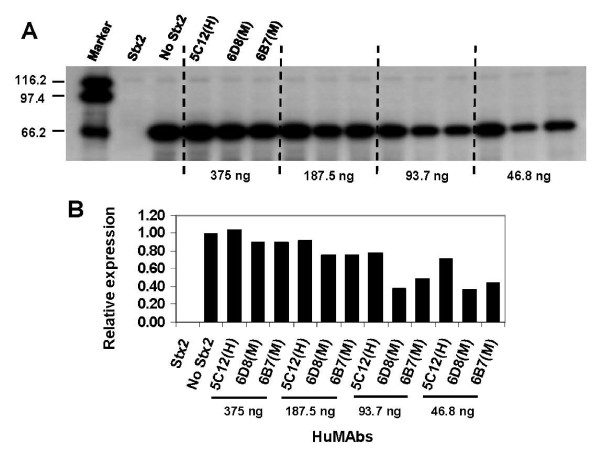
**Dose-dependent neutralization of the RNA-NGA of Stx2 by antibodies of high and medium HeLa cell cytotoxicity neutralization groups**. The letters 'H' or 'M' within parenthesis after a HuMAb designation represent high or medium HeLa cell cytotoxicity-neutralization group/s of the antibodies, respectively. The superiority of 5C12 over 6D8 and 6B7 to neutralize RNA-NGA of Stx2 was apparent at all doses, especially at the lower doses of 93.7 and 46.8 ng. At the 46.8 ng dose, the quantitative expression of the luciferase band in presence of the 5C12, 6D8 and 6B7 relative to when Stx2 was absent was 0.72, 0.37 and 0.44, respectively. The experiment was repeated once with similar results.

To validate the differences observed in luciferase translation (Fig. 1) in the presence of a single antibody dose between high and the medium Stx2-cytotoxicity neutralizing antibodies, dose response studies were performed utilizing 5C12 from the high neutralizing group and 6D8 and 6B7 from the medium neutralizing group (Fig. 2). The neutralizing activity of these antibodies was analyzed at different doses in the RNA N-glycosidase assay in the presence of 10 ng of Stx2. The superiority of 5C12 over 6D8 and 6B7 to neutralize RNA-NGA of Stx2 was apparent at all doses, especially at the lower doses of 93.7 and 46.8 ng (Fig. 2). At the 46.8 ng dose, the quantitative expression of the luciferase band in presence of the 5C12, 6D8 and 6B7 relative to when Stx2 was absent was 0.72, 0.37 and 0.44, respectively (Fig. 2B).

**Figure 3:**
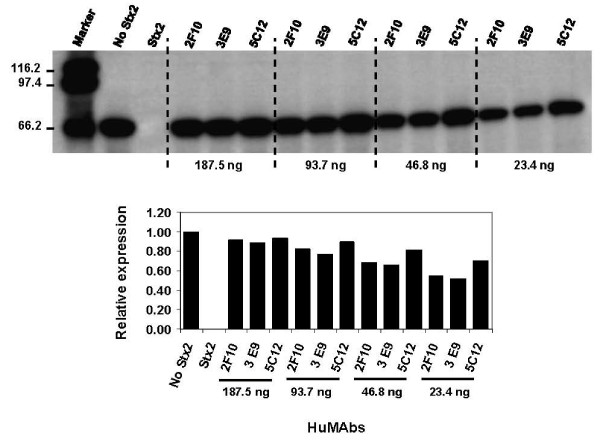
**Dose-dependent Neutralization of the RNA-NGA of Stx2 by the best HeLa cell cytotoxicity- and RNA-NGA-neutralizing HuMAbs 2F10, 3E9 and 5C12**. The differences in neutralizing efficiency were certainly apparent at the lower doses of 46.8 ng and 23.4 ng. At 46.8 ng dose, the quantitative expression of luciferase band in presence of 2F10, 3E9 and 5C12 relative to when Stx2 was absent was 0.68, 0.65 and 0.81, respectively. At 23.4 ng dose, the relative expression of luciferase band in presence of 2F10, 3E9 and 5C12 was 0.54, 0.51 and 0.70. The experiment was repeated once with similar results.

The dose response studies were also performed with the HuMAbs 2F10, 3E9, and 5C12 to determine the best RNA-NGA-neutralizing antibody (Fig. 3). The relative band intensities, especially at the doses of 46.8 ng and 23.4 ng, show that the intensities of the luciferase bands obtained with 2F10 and 3E9 were clearly lower than that of 5C12. This suggests that the neutralization of RNA-NGA by 5C12 was stronger than the other two antibodies.

### Neutralization of Stx2-induced mouse toxicity by the HuMAbs

The 19 HuMAbs utilized in this study have been evaluated before with the mouse toxicity model [20]. However, dose response studies were not performed (except for 5C12, 2F10 and 3E9), and antibodies produced in mouse ascites quantified by ELISA were used, as opposed to protein A-purified antibodies quantified by UV spectrophotometry used in the present study. Dose response studies were required in the present study to compare the relationship between neutralization of RNA-NGA of Stx2 and protection in vivo against Stx2 challenge. The dose response studies were performed on a few selected antibodies. The selection of these antibodies was based on their neutralizing activities in the RNA N-glycosidase assay. The HuMAbs 5C12 and 2F10 (strong or best RNA-NGA neutralizing antibodies), and 6D8 and 6B7 (moderate RNA-NGA neutralizing antibody), were selected. In addition, the HuMAb 6C3 was included since it had lesser RNA-NGA neutralizing activity than 6D8 and 6B7 (results of the present study), but protected mice against Stx2 challenge [20]. We evaluated the antibodies' activity in mice at the doses of 50 (3.5 mg/kg body weight) and 5 μg (350 μg/kg) per mouse.

**Figure 4:**
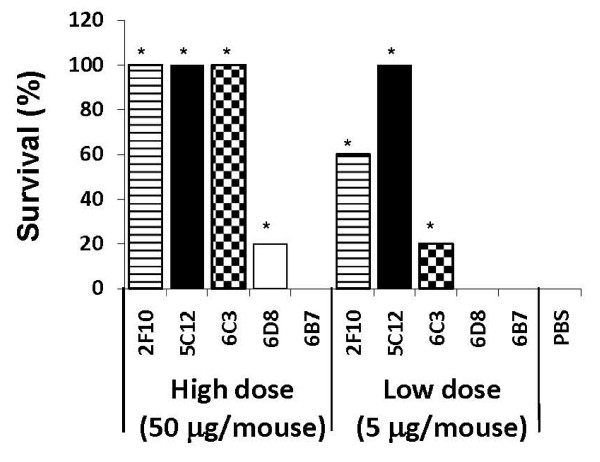
**Percent survival of mice given intraperitoneally (IP) 50 and 5 μg of HuMAbs 5C12, 2F10, 6B7, 6D8 and 6C3 followed 4 h later with IP administration of a lethal Stx2 dose**. Mice in PBS control groups died within 2 days of Stx2 challenge. Star (*) above the columns represents significant survival as compared to that of PBS. At 50 μg, 5C12, 2F10 and 6C3 completely protected mice against Stx2-mediated toxicosis and death. At the same dose, the antibodies 6B7 and 6D8 protected 0% and 20% mice, respectively. At the low dose of 5 μg, 5C12 was highly protective since it protected all mice, and 2F10 was moderately protective since it protected 60% of mice. At the same dose, the antibody 6C3 showed low level protection (protected 20% of mice), and the antibodies 6B7 and 6D8 failed to protect any mouse.

At 50 μg dose, 5C12, 2F10 and 6C3 completely protected mice against Stx2-mediated toxicosis and death (Fig. 4). At the same dose, the antibodies 6B7 and 6D8 protected 0% and 20% mice, respectively. The protection provided by 6D8 was barely significant (*p *= 0.04) to that provided by PBS. The HuMAbs 5C12, 2F10 and 6C3 provided significantly better protection than 6D8 (*p *= 0.01), 6B7 (*p *= 0.002) and PBS (*p *= 0.002). All mice in the PBS control group succumbed to Stx2 toxicity.

At the low dose of 5 μg, 5C12 was highly protective since it protected all mice, and 2F10 was moderately protective since it protected 60% of mice (Fig. 4), and they did not differ significantly from each other. The HuMAb 5C12 provided significantly better protection than 6C3 (*p *= 0.01), 6D8 (*p *= 0.002), 6B7 (*p *= 0.002) and PBS (*p *= 0.002). The protection provided by 2F10 was significantly better than 6D8 (*p *= 0.003), 6B7 (*p *= 0.003) and PBS (*p *= 0.003) but not significantly better than 6C3 (*p *= 0.2). Nonetheless, 2F10 protected 2.5 times more mice than 6C3. The antibody 6C3 showed low level protection (protected 20% of mice) which was significantly better than PBS (*p *= 0.01). The antibodies 6B7 and 6D8 failed to protect any mouse.

## Discussion

In the present study, we have demonstrated that ability of an A subunit-specific antibody to block the RNA-NGA of the toxin is directly related to its ability to neutralize Stx2-mediated HeLa cell death. Those antibodies that were better in blocking the RNA-NGA of Stx2 were also better in neutralizing Stx2 mediated HeLa cell death (Table 1 and Fig. 1). The Stx2A-specific HuMAbs 2F10, 3E9, and 5C12 were best in blocking the RNA-NGA of Stx2 as well as protecting HeLa cells against Stx2-mediated cytotoxicity. Furthermore, 5C12 was the strongest antibody in neutralizing both RNA-NGA (Fig. 3) and HeLa cell death mediated by Stx2 (Table 1). Those antibodies that neutralized RNA-NGA of the toxin lesser than 2F10, 3E9, and 5C12 also neutralized Stx2-mediated HeLa cell death lesser than these 3 antibodies, and those that neutralized RNA-NGA poorly (4H10, 5F2, 3A2, 6E6, 7F2 and 1G12) were similarly weaker in protecting HeLa cells against the toxin-death. The discrepancies between the present and an earlier study [20] in grouping of the HuMAbs based on the HeLa cell cytotoxicity neutralization abilities seem to be related to inconsistencies in quantification of proteins by ELISA in the previous study [20]. We utilized UV spectrophotometry in the present study since we observed that protein estimation by this method avoids variations due to assay plates and reagents that occur in ELISA and provides more accurate results.

However, with the exception of the best RNA-NGA blocking antibodies (5C12 and 2F10), the efficacies of antibody neutralization of RNA-NGA of Stx2 did not correlate with their *in vivo *protective efficacies. The HuMAbs 6D8 and 6B7 neutralized RNA-NGA of Stx2 moderately but failed to protect mice; 6B7 protected only 20% of mice at the highest dose of 50 μg/mouse against Stx2-death, and 6D8 failed to protect any mice at that dose. In contrast, 6C3 which neutralized RNA-NGA of Stx2 less effectively than 6D8 and 6B7 protected 100% mice at the same highest dose. At the 10 fold lower dose, 6C3 still protected 20% of mice whereas 6D8 and 6B7 did not protect any mice. The best RNA-NGA neutralizing antibodies 5C12 and 2F10 protected 100% and 60% of mice, respectively, at the 10 fold lower dose of 5 μg/mouse. These findings are in agreement with our earlier studies which showed that only 5C12, 2F10, 3E9 and 6C3, and none of the other A subunit-specific antibodies analyzed in the present study protected mice against Stx2 challenge [20]. The ability of the weak RNA-NGA neutralizing antibody 6C3 to protect mice strongly against Stx2 challenge suggest that neutralization of RNA-NGA of Stx2 by antibodies is not the only mechanism by which A subunit-specific antibodies can neutralize Stx2 in vivo. This argument is also strengthened by the failure of the moderate RNA-NGA neutralizing antibody 6B7 to protect any mouse. Another moderate RNA-NGA neutralizing antibody 6D8 protected only 20% mice at 50 μg/mouse dose and none at 5 μg/mouse dose.

It is not clear how RNA-NGA neutralizing activity of 5C12 neutralizes the toxicity of Stx2 for HeLa cells since our recent studies have shown that 5C12 blocks the retrograde transport of the toxin to TGN and ER, and prevents the Stx2 A subunit from entering the cytosol where it exerts its RNA-NGA [24]. It is possible that as a consequence of blocking RNA-NGA, 5C12 may mask the site of furin cleavage on the A subunit or induce conformational changes in the toxin such that the furin action on the A subunit is evaded, and therefore, the toxin remains intact and bound to Gb_3 _when inside the cell. Our studies have shown that the binding of 5C12 with the toxin does seem to induce conformational changes in the toxin molecule since binding affinity of Stx2 to the Gb3 receptor increases when Stx2 is bound by 5C12 than when it is unbound [24]. We have proposed that stronger binding of Stx2 with the Gb_3 _in presence of 5C12 may induce it to follow the same intracellular path as is followed by the Gb_3 _[24]. Although the intracellular passage of Gb3 is unknown, it is possible that Gb_3_, like transferrin receptor and some other host cell surface molecules, may be destined to be recycled back to the cell surface. However, a clear understanding of the mechanism by which 5C12 neutralizes Stx2 in vitro awaits further studies. In vivo clearance studies of 5C12/Stx2 immune complexes from our laboratory have recently shown that 5C12/Stx2 complexes are rapidly cleared from body, mostly by liver (manuscript in preparation).

Furin mediated cleavage of Shigella toxin A subunit is essential for efficient intoxication of cells [17]. Since Stx1 is identical to the Shigella toxin except a single amino acid change of serine at position 45 to a threonine, furin should also be essential for efficient catalytic activity of Stx1. However, Stx2 shares only 56% identity with Shigella toxin (or Stx1) at amino acid level [25]; consequently slight protein folding differences among these two Stx types make the catalytic site of the A subunit accessible in intact Stx2 [5]. This suggests that nicking of Stx2A by furin should not be essential for efficient RNA-NGA of Stx2, and that both Stx2A and Stx2A1 should have the same catalytic efficiency, as has been shown for a Stx2 variant [26]. In the present study, the holotoxin preparation consisted of the intact toxin and some of the Stx2A1 and Stx2A2 fragments (Western blot results, not shown). Such processing of Stx during purification by bacterial proteases is common and has been reported elsewhere [26]. However, presence of Stx2A1 does not affect its neutralization by 5C12 since 5C12 binds both Stx2A and Stx2A1 (Western blot results, not shown).

## Conclusion

In summary, the neutralization efficiency of the RNA-NGA of Stx2 by A subunit-specific antibodies correlate strongly with their abilities to protect HeLa cells against Stx2-mediated toxicity but only the strongest RNA-NGA-neutralizing antibodies correlate very well with both protecting HeLa cells and mice against Stx2 challenge.

## Methods

### Stx2

Stx2 was purified as described previously [27]. The Stx2 stock was dissolved at 50 μg/ml in phosphate buffered saline (PBS), aliquoted and stored at -20°C.

### Stx2-specific HuMAbs

Nineteen Stx2A-specific human monoclonal antibodies (HuMAbs) (1G1, 2F10, 3E9, 5C12, 6H5, 6H7, 7C4, 9H9, 14C12, 4H10, 5E12, 6C3, 6D8, 6E6, 1G12, 3A2, 5F2, 6B7, and 7F2) and one Stx2B-specific HuMAb (5H8) produced elsewhere [20] were included in the present study. All antibodies were of human IgG1 isotype. These antibodies were grouped as high, medium or low neutralizing antibodies based on their efficacies to neutralize Stx2 in a HeLa cell cytotoxicity assay [20]. The antibodies were quantified by ELISA [20]. However, we have observed that protein estimation by UV spectrophotometry provides more accurate results since variations due to assay plates and reagents that occur in ELISA are avoided. Since quantification by UV spectrophotometry requires purified protein, the antibodies were purified by protein A affinity chromatography, dialyzed against PBS, quantified by UV spectrophotometry (ND-1000 Spectrophotometer, Nanodrop), aliquoted, and stored at -20°C. The HuMAbs were quantified every time immediately before use in an experiment to ensure that their concentrations did not change.

### HeLa cell cytotoxicity assay

An *in vitro *HeLa cell cytotoxicity assay was performed to evaluate the ability of protein A-purified 19 HuMAbs to neutralize the cytotoxic effects of Stx2 as described elsewhere [23], and to group the antibodies as high, medium or low neutralizing. Briefly, Stx2 at 20 ng/ml concentration (killed > 80% of HeLa cells) was preincubated with the HuMAbs at 10.00 and 1.25 μg/ml for 1 h at 37°C in 5% CO_2_, and then added to the HeLa cells. The plates were incubated overnight at 37°C in 5% CO_2_. Dead cells were removed by washing with PBS, and viable cells stained with Crystal Violet. The absorbance was read at 690 nm, and the percent neutralization of Stx2-mediated HeLa cell cytotoxicity by the HuMAbs was calculated by the formula: [(OD_toxin + HuMAb _- OD_toxin only_)/(OD_no toxin _- OD_toxin only_)] × 100, where the ODs included from the wells containing toxin plus HuMAb (OD_toxin + HuMAb_), toxin only (OD_toxin only_), and no toxin or cell culture medium only (OD_no toxin_) [23]. The experiments were repeated three times and each antibody dose was tested in duplicate wells.

For statistical analysis, comparisons of the means of neutralization rates (%) were made among 19 HuMAbs at 3 different concentrations of antibodies using analysis of variance (ANOVA) test. Pairwise multiple comparisons were then done using Fisher LSD Method. Resulting *p*-values of less than 0.05 were considered significant.

### In vitro translation in a rabbit reticulocyte lysate system

*In vitro *translation system utilizing rabbit reticulocyte lysate [15] and biotinylated lysine tRNA (Transcend™ tRNA) was used to measure RNA-NGA of holotoxin Stx2. The RNA NGA site of the A subunit is accessible in the intact holotoxin Stx2 [5]. All reagents, including the Flexi rabbit reticulocyte lysate kit, were purchased from Promega (Madison, WI). Protein translation conditions were standardized by adjusting the concentrations of each component, especially potassium, magnesium, and transcend™ tRNA. The final translation reactions, assembled in conical-bottom 96-well plates at a final volume of 50 μl per sample, consisted of 10 μl of basic components (Nuclease-free water, 2.5 M potassium chloride, 25 mM magnesium acetate, 40 units/μl Rnasin^®^ribonuclease inhibitor, 1 mM complete amino acid, transcend™ tRNA, and 1 mg/ml luciferase template RNA), 24 μl of rabbit reticulocyte lysate, and 16 μl of the samples (PBS or Stx2 or Stx2 plus HuMAbs).

To quantify inhibition of protein synthesis by Stx2, a stock Stx2 solution was serially diluted (40 to 0.31 ng of Stx2 in 16 μl volume) in PBS and tested in the rabbit reticulocyte lysate for inhibition of translation of luciferase. A 16 μl aliquot of Stx2 was incubated with 24 μl of reticulocyte lysate at room temperature (RT) for 30 min. Ten μl of basic components were then added, and the plates incubated for 1 h at 30°C in a waterbath. The reaction was terminated by placing the plates on ice, and the biotinylated luciferase analyzed by Western blotting.

To investigate the ability of 19 Stx2A-specific HuMAbs to neutralize the Stx2-mediated inhibition of protein synthesis, a dose of 10 ng of Stx2 was selected since this dose inhibited protein synthesis completely, as determined from studies outlined above. Human IgG1 isotype (Sigma), and a HuMAb 5H8 against the B subunit of Stx2, were included as controls. For these experiments, 10 ng of Stx2 in 8 μl PBS were incubated with 93.7 ng of Stx2-specific HuMAbs in 8 μl PBS at RT for 30 min. The rest of the steps were same as described above, and the translated luciferase protein was again analyzed by Western blotting. The concentration of 93.7 ng utilized in these studies was based on a few dose response studies conducted on selected antibodies to determine an antibody dose that would strongly neutralize the RNA-NGA of 10 ng Stx2.

### Western blot analysis

The translated biotinylated luciferase was separated by sodium dodecyl sulfate polyacrylamide gel electrophoresis (SDS-PAGE) under reducing conditions, and electrophoretically transferred to a PVDF membrane (Bio-Rad). After washing with TBS-T (Tris-buffered saline, 0.05% Tween^® ^20), the membrane was incubated at RT for 30 min with streptavidin-HRP conjugate (Promega, Madison, WI) diluted 1:30,000 in TBS-T. After washing, the membrane was incubated with a chemiluminescent substrate (ECL plus western blotting detection system, Amersham Biosciences), and exposed to Kodak BioMax film (VWR international, Bridgeport, NJ) for 1-5 min. The bands were scanned by Kodak Image Station 2000RT and their intensities analyzed with Kodak 1D Image Analysis Software version 3.6.5 K2. Luciferase band intensities in presence of antibodies were calculated relative to the band intensities when Stx2 was absent.

### Mouse protective efficacy

The mouse toxicity model was used to determine protective efficacy of selected HuMAbs against a lethal Stx2 dose in vivo as described elsewhere [23]. Groups of five 3- to 4-week-old female Swiss Webster mice (Taconic) were injected intraperitoneally (IP) with 50 μg/mouse or 5 μg/mouse of antibody, or PBS, 4 hr prior to the IP administration of a lethal Stx2 dose (75 ng/mouse in 200 μl of PBS). Mice were observed 3 or more times daily for clinical signs and survival. All mouse procedures were approved by the Tufts University Institutional Animal Care and Use Committee.

For statistical analysis, the grouped survival data was analyzed by applying Mantel-Cox test and performed using PROC Frequency procedure of statistical software SAS. Resulting *p*-values of less than 0.05 were considered significant.

## Competing interests

The authors declare that they have no competing interests.

## Abbreviations

Stx: Shiga toxin; STEC: Stx-producing *Escherichia coli*; HUS: hemolytic uremic syndrome; Gb_3_: globotriaosyl ceramide; Gb_4_: globotetraosylceramide; TGN: trans-Golgi network; ER: endoplasmic reticulum; HuMAbs: human monoclonal antibodies; RNA-NGA: RNA *N*-glycosidase activity; STEC: Stx-producing *Escherichia coli*; HUS: hemolytic uremic syndrome.

## Authors' contributions

KJ carried out most of the assays, analyzed data, performed some statistical analysis, participated in the design of the study and helped with draft of the manuscript. SC purified antibodies and helped with the HeLa cell cytotoxicity assay. PS performed statistical analysis. JL helped with standardization of the *in vitro *translation assay. ST helped with draft of the manuscript. AS conceived and designed the study, analyzed data, and drafted the manuscript. All authors read and approved the final manuscript.
